# Supplementary material for: Ubiquitination-dependent control of sexual differentiation in fission yeast
Source: eLife. 2017 Aug 25;6:e28046. doi: 10.7554/eLife.28046 (PMC5614563; doi:10.7554/eLife.28046)
Supplement: Supplementary file 1. [file elife-28046-supp1.docx]

**Supplementary file 1. *S. pombe* strains used in this study**

| Strain | Genotype | Source |
| --- | --- | --- |
| PR040 | h90, *ura4-DS/E, ade6-M210, leu1-32, mat3M::ura4+* | D. Moazed |
| PR167 | h90, *ura4-DS/E, leu1-32, mat3M::gfp+::nat^R^MX* | M. Bühler |
| PR206 | PR167, rrp6::*kan^R^MX* | This study |
| PR314 | PR040, Mmi1-TAP::*hph^R^MX* | This study |
| PR389 | PR040, Caf1-3xFLAG::*kan^R^MX* | This study |
| PR398 | PR040, Not1-3xFLAG::*kan^R^MX* | This study |
| PR403 | PR040, Mmi1-TAP::*hph^R^MX* Caf1-3xFLAG::*kan^R^MX* | This study |
| PR404 | PR040, Mmi1-TAP::*hph^R^MX* Not1-3xFLAG::*kan^R^MX* | This study |
| PR425 | PR040, caf1::*nat^R^MX* | This study |
| PR426 | PR040, ccr4::*nat^R^MX* | This study |
| PR430 | PR040, Mtl1-TAP::*hph^R^MX* Not1-3xFLAG::*kan^R^MX* | This study |
| PR440 | PR040, red1::*nat^R^MX* Mmi1-TAP::*hph^R^MX* Not1-3xFLAG::*kan^R^MX* | This study |
| PR446 | PR040, Red1-TAP::*hph^R^MX* Not1-3xFLAG::*kan^R^MX* | This study |
| PR469 | PR040, not3::*kan^R^MX* | This study |
| PR471 | PR040, rcd1::*kan^R^MX* | This study |
| PR483 | PR040, not2::*nat^R^MX* | This study |
| PR486 | PR040, rrp6::*hph^R^MX* | This study |
| PR523 | PR040, mot2::*nat^R^MX* Mmi1-TAP::*hph^R^MX* | This study |
| PR524 | PR040, mot2::*nat^R^MX* Caf1-3xFLAG::*kan^R^MX* | This study |
| PR526 | PR167, mot2::*kan^R^MX* | This study |
| PR576 | PR040, Mot2-3xFLAG::*kan^R^MX* | This study |
| PR597 | PR040, *kan^R^MX*::P41nmt1-3xFLAG-Mmi1 | This study |
| PR637 | PR040, Mei2-3xHA::*hph^R^MX* | This study |
| PR646 | PR040, *kan^R^MX*::P41nmt1-TAP-Mmi1 Mei2-3xHA::*hph^R^MX* | This study |
| PR647 | PR040, mot2::*nat^R^MX* Mei2-3xHA::*hph^R^MX* | This study |
| PR652 | PR040, mei2::*hph^R^MX* | This study |
| PR657 | PR040, mot2::*nat^R^MX* mei2::*hph^R^MX* | This study |
| PR658 | PR040, rrp6::*nat^R^MX* mei2::*hph^R^MX* | This study |
| PR662 | PR040, pREP1-6His-Ubi::*LEU2* | This study |
| PR664 | PR040, mot2::*nat^R^MX* *kan^R^MX*::P41nmt1-TAP-Mmi1 Mei2-3xHA::*hph^R^MX* | This study |
| PR667 | PR040, Mei2-3xHA::*hph^R^MX*, pREP1-6His-Ubi::*LEU2* | This study |
| PR668 | PR040, mot2::*nat^R^MX* Mei2-3xHA::*hph^R^MX*, pREP1-6His-Ubi::*LEU2* | This study |
| PR669 | PR040, *kan^R^MX*::P3nmt1-3xFLAG-Mei2 | This study |
| PR675 | PR040, *kan^R^MX*::P41nmt1-TAP-Mei2 | This study |
| PR676 | PR040, Mei2-GFP::*kan^R^MX* | This study |
| PR687 | PR040, mot2::*nat^R^MX* Mei2-GFP::*kan^R^MX* | This study |
| PR714 | PR040, mei4::*nat^R^MX* *kan^R^MX*::P41nmt1-TAP-Mmi1-(1-65)∆ Mei2-3xHA::*hph^R^MX* | This study |
| PR720 | PR040, mot2::*nat^R^MX* *kan^R^MX*::P41nmt1-TAP-Mei2 | This study |
| PR721 | PR040, red1::*nat^R^MX* *kan^R^MX*::P41nmt1-TAP-Mei2 | This study |
| PR722 | PR040, caf1::*nat^R^MX* *kan^R^MX*::P41nmt1-TAP-Mei2 | This study |
| PR723 | PR040, rrp6::*nat^R^MX* *kan^R^MX*::P41nmt1-TAP-Mei2 | This study |
| PR725 | h-, *ura4-D18, leu1-32, mts2-1* Mei2-3xHA*::hph^R^MX* | This study |
| PR726 | PR040, mei4::*nat^R^MX* mmi1::*hph^R^MX kan^R^MX*::P41nmt1-TAP-Mei2 | This study |
| PR728 | h-, *ura4-D18, leu1-32, mts2-1* mot2::*nat^R^MX* Mei2-3xHA*::hph^R^MX* | This study |
| PR731 | PR040, mot2::*nat^R^MX* *kan^R^MX*::P41nmt1-TAP-Mei2 pREP41::*LEU2* | This study |
| PR735 | PR040, ccr4::*nat^R^MX* *kan^R^MX*::P41nmt1-TAP-Mei2 | This study |
| PR736 | PR040, not2::*nat^R^MX* *kan^R^MX*::P41nmt1-TAP-Mei2 | This study |
| PR737 | PR040, not3::*nat^R^MX* *kan^R^MX*::P41nmt1-TAP-Mei2 | This study |
| PR747 | h-, *ura4-D18, leu1-32, mts2-1,* Mei2-3xHA*::hph^R^MX* pREP1-6His-Ubi::*LEU2* | This study |
| PR748 | h-, *ura4-D18, leu1-32, mts2-1* mot2::*nat^R^MX* Mei2-3xHA*::hph^R^MX* pREP1-6His-Ubi::*LEU2* | This study |
| PR753 | PR040, Mot2-GFP::*kan^R^MX* | This study |
| PR759 | PR040, red1::*nat^R^MX* Mmi1-TAP::*hph^R^MX* Caf1-3xFLAG::*kan^R^MX* | This study |
| PR785 | PR040, Mmi1-TAP::*hph^R^MX* Mot2-GFP::*kan^R^MX* | This study |
| PR790 | PR040, red1::*nat^R^MX* Mmi1-TAP::*hph^R^MX* Mot2-GFP::*kan^R^MX* | This study |
| PR792 | PR040, mot2::*nat^R^MX* | This study |
| PR802 | PR040, mot2::*nat^R^MX* Mmi1-TAP::*hph^R^MX* Mei2-GFP::*kan^R^MX* | This study |
| PR821 | PR040, ubr1::*kan^R^MX*, Mei2-3xHA*::hph^R^MX* | This study |
| PR853 | PR040, ubr1::*kan^R^MX* Mei2-3xHA*::hph^R^MX* pREP1-6His-Ubi::*LEU2* | This study |
| PR860 | PR040, rcd1::*hph^R^MX* *kan^R^MX*::P41nmt1-TAP-Mei2 | This study |
| PR869 | PR040, ubr1::*hph^R^MX* | This study |
| PR882 | PR040, Tef3-3xFLAG::*kan^R^MX* | This study |
| PR883 | PR040, Rpl1601-3xFLAG::*kan^R^MX* | This study |
| PR884 | PR040, *kan^R^MX*::P3nmt1-3xFLAG-Mei2 pREP1-6His-Ubi::*LEU2* | This study |
| PR887 | PR040, mot2::*nat^R^MX* Rpl1601-3xFLAG::*kan^R^MX* | This study |
| PR888 | PR040, mot2::*nat^R^MX* Tef3-3xFLAG::*kan^R^MX* | This study |
| PR889 | PR040, mot2::*nat^R^MX* *kan^R^MX*::P41nmt1-TAP-Mei2 pREP41-Mot2-2xFLAG::*LEU2* | This study |
| PR890 | PR040, mot2::*nat^R^MX* *kan^R^MX*::P41nmt1-TAP-Mei2 pREP41-Mot2-RING∆-2xFLAG::*LEU2* | This study |
| PR893 | PR040, ubr1::*hph^R^MX* Mei2-GFP::*kan^R^MX* | This study |
| PR904 | PR040, mot2::*nat^R^MX* *kan^R^MX*::P41nmt1-TAP-Mei2 pREP41-Mot2-C37A-2xFLAG::*LEU2* | This study |
| PR905 | PR040, mot2::*nat^R^MX* *kan^R^MX*::P41nmt1-TAP-Mei2 pREP41-Mot2-C45A-2xFLAG::*LEU2* | This study |
| PR906 | PR040, mot2::*nat^R^MX* *kan^R^MX*::P41nmt1-TAP-Mei2 pREP41-Mot2-C57A-2xFLAG::*LEU2* | This study |
| PR930 | PR040, Mei2-3xFLAG::*kan^R^MX* pREP1-6His-Ubi::*LEU2* | This study |
