# Supplementary material for: Ubiquitination-dependent control of sexual differentiation in fission yeast
Source: eLife. 2017 Aug 25;6:e28046. doi: 10.7554/eLife.28046 (PMC5614563; doi:10.7554/eLife.28046)
Supplement: Supplementary file 2. [file elife-28046-supp2.docx]

**Supplementary file 2.** **Oligonucleotides used in this study**

| Primers | Sequence | Related figures |
| --- | --- | --- |
| P249: *mei4+* fwd | 5’-TGGATCAGATCCGTGGAATC-3’ | 2A-B, S2, 4A-B, 5C, S1A, 6D |
| P250: *mei4+* rev | 5’-AACGCTCGATTAGAAGGCAT-3’ | 2A-B, S2, 4A-B, 5C, S1A, 6D |
| P253: *act1+* fwd | 5’-AACCCTCAGCTTTGGGTCTT-3’ | 2A-B, S2, 3D, 4A-B, 5A-C, S1A, S3A, S5A-B, 6D |
| P254: *act1+* rev | 5’-TTTGCATACGATCGGCAATA-3’ | 2A-B, S2, 3D, 4A-B, 5A-C, S1A, S3A, S5A-B, 6D |
| P325: *ssm4+* fwd | 5’-ACACAGTTTACGGGATTCTA-3’ | 2A-B, 3D, 4A-B, S1A, 6D |
| P326: *ssm4+* rev | 5’-GATTGTGATGAAAACTGGGT-3’ | 2A-B, 3D, 4A-B, S1A, 6D |
| P607: *mcp5+* fwd | 5’-AGACGTATTCACCTTACCTC-3’ | 2A-B, S2, 3D, 4A-B, 5C, S1A, 6D |
| P608: *mcp5+* rev | 5’-GTTTCCCATCATGACATGTT-3’ | 2A-B, S2, 3D, 4A-B, 5C, S1A, 6D |
| P645: *sme2+* fwd | 5’- TTGCCGATTTCACGAAGTT-3’ | 4A-B |
| P646: *sme2+* rev | 5’- ATCTGTCTGTTCTGCTGCT-3’ | 4A-B |
| P855: *mei2+* fwd | 5’-CCAACAAGGGTACCTATGAT-3’ | 3D, S3A, 6D |
| P882: *mei2*+ rev | 5’-GAGTACCCACTCTAGCTTTG-3’ | 3D, S3A, 6D |
| P1010: *mei2+* fwd | 5’-GAGTTGGTGAACGGAAAGTA-3’ | 5A-C, S5A-B |
| P881: *mei2*+ rev | 5’-GGGATTCTGAGAGAACAGAA-3’ | 5A-C, S5A-B |
